# Liver Cirrhosis Caused by Food‐Borne Zoonotic *Fasciola gigantica* in Cattle in Bangladesh: Pathology and Immunological Orchestra

**DOI:** 10.1002/iid3.70320

**Published:** 2026-01-08

**Authors:** Md. Haydar Ali, Joydeep Paul, Romana Parvin, Md. Shahadat Hossain, Sharmin Shahid Labony, Nusrat Nowrin Shohana, Md. Mahmudul Alam, Umme Razia Islam, Anita Rani Dey, Md. Abu Hadi Noor Ali Khan, Md. Abdul Alim, Anisuzzaman

**Affiliations:** ^1^ Department of Parasitology Bangladesh Agricultural University Mymensingh Bangladesh; ^2^ Department of Pathology and Parasitology Hajee Mohammad Danesh Science and Technology University Dinajpur Bangladesh; ^3^ Department of Biotechnology, School of Life Science and Biotechnology Adamas University Kolkata India; ^4^ Department of Surgery and Theriogenology Bangladesh Agricultural University Mymensingh Bangladesh; ^5^ Department of Pathology Bangladesh Agricultural University Mymensingh Bangladesh

**Keywords:** cytokines, fasciola gigantica, fasciolosis, hepatic pathology, liver cirrhosis, Th2 immune response, transcription factors

## Abstract

**Background:**

Fasciolosis is a food‐borne parasitic zoonotic disease caused by widespread liver flukes that affect ruminants and humans, and is responsible for non‐resolving hepatic damage. Although fasciolosis occurs in both acute and chronic forms, chronic fasciolosis is more common.

**Objectives:**

This study investigates the pathological changes and immunological cascade in the livers of *Fasciola gigantica* infected cattle, both at the transcriptional and translational levels.

**Methods:**

Normal and suspected liver samples from cattle were collected and examined. Affected tissues were subjected to routine histological and immunohistochemical analysis. Transcription factors and interleukins (IL) were measured by sqRT‐PCR and ELISA.

**Results:**

In chronic fasciolosis, liver became atrophied, marked with whitish fibrotic patches, calcification and bile duct hyperplasia filled with blackish‐brown sandy contents. Histopathological examinations showed massive loss of hepatocytes, fibrous proliferation, and infiltration of mononuclear cells and eosinophils. In addition, a huge deposition of amyloid was noticed in severely affected livers. T‐bet (T‐box expressed in T cells protein) remained at the basal level, whereas expression of GATA‐3 (GATA‐binding protein 3) was dramatically increased in severe fasciolosis. Also, there was a noticeable increase in the GATA‐3 positive cells, while T‐bet positive cells were largely absent, displaying a drift towards Th2 type immune response. In severe infection, mRNAs of IL‐4, IL‐5, IL‐6, IL‐10 and IL‐13 highly expressed compared to the non‐infected control, but the interferon‐ (INF)‐γ expression remained unaltered. Similarly, we detected significant (*p* < 0.01) elevation of IL‐4, IL‐5, IL‐6 and IL‐13 in severely affected liver lysate. To further validate the notion, bovine peripheral blood mononuclear cells (bPBMCs) were treated with *F. gigantica‐*culture milieu (FCM). FCM treatment elevated IL‐4, IL‐5, IL‐6 and IL‐13 in a time dependent manner, confirming liver fluke‐induced Th2‐biased immune response.

**Conclusion:**

This study reveals distinct pathology and dysregulation of transcription factors and cytokines profiles in *F. gigantica* infected cattle.

## 1 Introduction

Fasciolosis is a food and water‐borne parasitic zoonotic disease caused by *Fasciola hepatica* and *Fasciola gigantica* (Trematoda: Fasciolidae, commonly called liver flukes), distributed on every inhabited continent, except Antarctica [1]. Of the two fasciolid flukes, *F. hepatica* is mainly prevalent in temperate regions but is also recorded in tropical and subtropical countries. Conversely, *F. gigantica* is predominantly found in tropical regions of Asia, the Middle East and Africa. However, a sterile hybrid population is often reported in some regions where both species co‐exist [2, 3, 4]. Furthermore, parthenogenetic *Fasciola* which is capable to reproduce without fertilization, is spreading very rapidly throughout the globe [5, 6]. Fasciolosis is the third most important parasitic disease in the livestock industry, infecting millions of ruminants worldwide and associated with huge economic loss, estimated over US$ 3.2 billion annually [7, 8]. The World Health Organization (WHO) has listed the malady as a neglected tropical disease (NTD) since an estimated 17 million people have already been infected, and a further 180 million people are at risk [9, 10, 11].

The geographical distribution and global prevalence of bovine fasciolosis ranges from 1.2% to 91.0% in Africa, 3.0%–66.7% in America, 0.71%–69.2% in Asia, 26.5%–81.0% in Oceania and 0.12%–86.0% in Europe [8]. Previous published reports revealed that the prevalence of fasciolosis in cattle in Bangladesh is 21%–53% [12, 13]. In Bangladesh, fasciolosis has been detected in goats (26.11%) and sheep (24.00%) [14]. However, Ahasan et al (2023) [15] reported the prevalence of fasciolosis as 35.4%, 26.8%, 55.5%, and 29.3% in goats, sheep, buffaloes and cattle, respectively in the country. On the other hand, Shykat et al (2022) [12] found that 35.4% goats are infected with fasciolosis in the Northern part of Bangladesh. Through coprological examination, fasciolosis has been detected in 39.5% small ruminants in the middle part of the country [16]. Even fasciolosis has been detected in livestock in Saint Martin's Island, the southeastern offshore area of Bangladesh in the Bay of Bengal [17], suggesting the disease is prevalent throughout the country.

Following the ingestion of metacercariae (the infective stage), the newly excysted juveniles (NEJs) are released and reached the liver. NEJs migrate through the liver parenchyma and enter the bile ducts, where they become mature [18, 19]. During migration, NEJs destroy liver parenchyma, resulting in hemorrhagic black tunnels in the liver [20, 21]. Chronic form of fasciolosis is caused by the adult flukes, which is characterized by cirrhosis, atrophy, thickening and calcification of the bile ducts. Chronic fasciolosis is clinically manifested by hypoproteinemia, anemia, edema, stunted growth, weight loss, bottle jaw and often death [9, 22].

Balanced hepatic immuno‐inflammatory mechanisms are essential to maintain liver homeostasis and if disrupted (e.g. due to parasite infection), can lead to liver pathology and dysfunction [23]. Moreover, the abnormal increase of Th2 cytokines and/or transcription factors can also limit the control of *Fasciola* infection [24, 25]. During the early stages of infection, *F. hepatica* induces a mixed Th1/Th2 response characterized by up‐regulating IL‐4, IL‐10, and TGF‐b. As the infection proceeds, a predominant Th2 response is initiated along with the suppression of Th1‐based inflammation [26]. Transcription factors such as T‐bet (T‐box expressed in T cells proteins) and GATA‐3 (GATA‐binding protein 3) also play important roles in the differentiation of Th1 and Th2, respectively to regulate the production of cytokines [27, 28]. Liver flukes try to bypass the ongoing host immune responses to neutralize any possible pathological response; thus, ensure the survival of the parasites within the hosts [29]. Although immunological impairment and polarization of the Th1/Th2 balance are major consequences of *Fasciola*‐induced liver pathologies, the expression profile and dynamics of Th1/Th2 cytokines during *F. gigantica* infection have not yet been elucidated in naturally infected cattle. Here, we demonstrate hepatic pathologies induced by liver flukes and immunological consequences at both transcriptional and translational levels in the livers of cattle, naturally infected with *F. gigantica*.

## 2 Materials and Methods

### 2.1 Sampling, Parasite Collection and Identification

Liver samples were collected from the slaughterhouses located at Mymensingh (Co‐ordinates: 24°38′3″ N 90°16′4″ E) and Madhupur (Co‐ordinates: 24° 37’ 0.12“N 90° 01’ 30.00“E) areas of Bangladesh. Immediately after slaughtering, livers were grossly examined by close inspection and digital palpation for the presence of lesions produced by liver flukes. Then the livers were dissected through the course of bile ducts and flukes were isolated. Isolated flukes were washed and identified by preparing permanent slides following the keys and description given by Itagaki et al [5].

To confirm the species, genomic DNA was isolated from flukes using the DNeasy Blood and Tissue kit following the manufacturer's instructions (Qiagen, Hilden, Germany). Briefly, only the cephalic cone area of a fluke was collected and homogenized. Tissues were treated with the cell lysis buffer and protease K, and centrifuged. Supernatant was collected and passed through DNA purification column and entrapped DNA was eluted using elution buffer. To validate the morphometry‐based identification, previously established *pepck* (phosphoenolpyruvate carboxykinase) ‐based multiplex PCR was conducted using specific primers (Fh‐Pepck‐F: GAT TGC ACC GTT AGG TTA GC; Fg‐Pepck‐F: AAA GTT TCT ATC CCG AAC GAA G; Fcmn‐Pepck‐R: CGA AAA TTA TGG CAT CAA TGG G). The PCR was conducted in a total volume of 25 μL, which contained 12.5 μL of master mix (OneTaq® Quick Load), 1 μL of 10 pmol of each forward primer (Fh‐pepck‐F and Fg‐pepck‐F), 2 μL of 10 pmol of common reverse primer (Fcmn‐pepck‐R) and 100 ng of gDNA. PCR cycles consisted of an initial denaturation at 94°C for 1.5 min, followed by 30 cycles at 94°C for 30 s, 61°C for 30 s and 72°C for 1 min with a final extension at 72°C for 10 min [5, 30].

### 2.2 Sample Collectihon, Preservation and Histopathology

Suspected parts of the affected liver or normal livers were collected and trimmed into small pieces. Infected/non‐infected control tissues were preserved in Carnoy's solution (a fixative containing absolute ethanol and glacial acetic acid, 3:1), and subjected to histopathological assessment using Hematoxylin and Eosin (H&E), Congo red (CR) and Masson's Trichrome (MT) stains. Also, infected/non‐infected control tissues were preserved in RNA later™ (Invitrogen, Thermo Fisher Scientific, MA, USA) or PBS at −80°C for total RNA or protein extraction.

### 2.3 Immunohistochemistry

Thin sections (5 µm) were prepared from the infected and non‐infected preserved tissues. Immunohistochemistry was performed for GATA‐3 and T‐bet using GATA‐3 monoclonal antibody (Invitrogen, Rockford, USA) and T‐bet monoclonal antibody (Invitrogen, Bengaluru, India) following the previously described protocol [31, 32].

### 2.4 Total RNA (tRNA) Extraction and Complementary DNA (cDNA) Synthesis

RNA later™ preserved tissues were homogenized in liquid nitrogen and total RNA was collected using RNeasy Mini Kit following the manufacturer's instructions (Qiagen, Germany), and the concentration of the extracted RNA was measured using a Nanodrop^TM^ spectrophotometer. Thereafter, cDNA was synthesized *in vitro* using cDNA Synthesis Kit (Super Script™ III, Invitrogen, Thermo Fisher Scientific, MA, USA). Then concentration of cDNA was estimated using a nanodrop spectrophotometer (NanoDrop microvolume spectrophotometers™, Thermo Fisher Scientific, MA, USA) and kept at −20°C.

### 2.5 Detection of Cytokines and Transcription Factors by sqRT‐PCR and Electrophoresis

Prepared cDNA was used to detect the level of expression of several cytokines (IFN‐γ, IL‐4, IL‐5, IL‐6, IL‐10, and IL‐13) and transcription factors (T‐bet and GATA‐3) by performing sqRT‐PCR with specific primers (Table 1). The glyceraldehyde‐3‐phosphate dehydrogenase (GAPDH) was used as the internal control. PCR was performed in a total volume of 25 μL with 12.5 μL master mixture (OneTaq® Quick Load Polymerase, BioLab, USA) containing polymerase enzyme, dNTP and MgCl_2_, 1 μL of 10 pmol of each primer and 100 ng of gDNA (Table 1). The PCR products were analyzed on 1.5% agarose gel and visualized by ethidium bromide as described previously [39]. The level of expression of each gene was normalized using GAPDH as a housekeeping gene. Band thickness was analyzed using the ImageJ software. The value was expressed in an arbitrary unit (AU).

**Table 1:** Specific primers targeting cytokines and transcription factors.

Genes	Primers	Sequences (5′–3΄)	Annealing (°C/Sec)	Elongation (°C/Sec)	Amplicon (bp)	References
IFN‐γ	IFN‐gF	GCC AAA TTG TCT CCT TCT ACT TC	61°C/7	72°C/22	484	[33]
	IFN‐gR	GGG TCA AGT GAA ATA GTC ACA GG				
IL‐4	IL‐4F	TGC ATT GTT AGC GTC TCC TG	56 C/6	72°C/17	450	
	IL‐4R	AGG TCT TTC AGC GTA CTT GT				
IL‐5	IL‐5F	AGGTGATGGGAACTTGATGATT	59°C/20	72°C/20	116	[34]
	IL‐5R	CAGCATCCCCTTGTGCAGT				
IL‐6	IL‐6F	AAT GAG AAA GGA GAT ATG TGA GAA	58°C/30	72°C/30	405	[35]
	IL‐6R	CTG AAC TGC AGG AAA TTC TCA AGG				
IL‐10	IL‐10F	CTT TAA GGG TTA CCT GGG TTG C	60°C/30	72°C/45	239	[36]
	IL‐10R	CTC ACT CAT GGC TTT GTA GAC AC				
IL‐13	IL‐13F	GGT GGC CTC ACC TCC CCA AG	60°C/7	72°C/9	234	[33]
	IL‐13F	GAT GAC ACT GCA GTT GGA GAT GCT G				
T‐bet	T‐bet‐F	CGG CTG CAT ATC GTT GAG GT	55°C/45	72°C/45	107	[37]
	T‐bet‐R	GTC CCC ATT GGC ATTCCT C				
GATA‐3	GATA‐3F	CCA GAC CAG AAA CCG AAA AA	62°C/10	72°C/30	234	[38]
	GATA‐3R	ACC ATA CTG GAA GGG TGG TG				
GAPDH	GAP‐F	GGC GTG AAC CAC GAG AAG TAT AA	59°C/7	72°C/5	194	[33]
	GAP‐R	CCC TCC ACG ATG CCA AAG T				

### 2.6 Total Protein Extraction From Infected Liver Tissues

Tissues preserved in PBS were macerated using a pestle and mortar. Then the grinded tissues were sonicated and total protein (liver lysate) was extracted following the procedures described previously [40].

### 2.7 *Ex vivo* Culture of Adult Liver Flukes

Adult *F. gigantica* was isolated by systemic dissection of the liver along the course of the bile ducts, washed in PBS and then with DMEM (Sigma‐Aldrich, Darmstadt, Germany) supplemented with 200 U/mL penicillin and 200 mg/mL streptomycin (Sigma‐Aldrich, Germany). Adult flukes (2 parasites in 2 mL) were incubated in DMEM (Sigma‐Aldrich, Germany), supplemented with 20% bovine serum (BS, GE Healthcare Life Science Hyclone Laboratories, Utah, USA) along with 200 U/mL penicillin and 200 mg/mL streptomycin (Sigma‐Aldrich, Germany) in a 12‐well flat bottom cell culture plate (Corning Incorporated, NY, USA). Flukes were incubated at 37°C in 5% CO_2_ in a humidified air for 24 h. *Fasciola* culture media (FCM) was collected and centrifuged at 14,000 rpm for 5 min. Supernatant was harvested and kept at −20°C.

### 2.8 Bovine Peripheral Mononuclear Cells (bPBMCs) Isolation and Stimulation With FCM

Blood samples were collected from liver flukes‐naive cattle of 1‐year‐old in EDTA‐treated tubes. bPBMCs were isolated using Ficoll‐Paque^TM^ (GE Healthcare Bio‐Science AB, Uppsala, Sweden). Cells were washed in PBS and counted. Then 10^5^ bPBMCs were plated in a flat‐ bottom 96‐well cell culture plate in RPMI media (Sigma Aldrich, Germany) in a total volume of 100 μL supplemented with 200 U/mL penicillin and 200 mg/mL streptomycin (Sigma‐Aldrich, Germany) in the same condition as mentioned above. Cells were stimulated with 100 μL of FCM for 24 h. Unstimulated cells served as control. Cell culture media were collected at the indicated time points (1–24 h).

### 2.9 Sandwich ELISA

Harvested total protein (liver lysate) and cell culture media was analyzed by sandwich ELISA to detect IL‐4, IL‐5, IL‐6, IL‐13, and IFN‐γ following the manufacturer's protocol of commercially available ELISA kits (Catalog Number: EB12RB for bovine IL‐4, ESS0029 for bovine IL‐6, EB9RB for bovine IL‐13 and ESS0026B for bovine IFN‐γ; Invitrogen, Thermo Fisher Scientific, MA, USA, and Catalog Number: EB0172, Optics Valley Biopharmaceutical Accelerator, Wuhan, 430074, Hubei, China for bovine IL‐5).

### 2.10 Statistical Analysis

All collected data were encoded into a Microsoft Excel spreadsheet. Statistical analyses were performed using IBM SPSS version 22. Data were presented as mean ± SE. Variables were compared using *χ* ^2^ test and *p* < 0.05 was considered as statistically significant.

## 3 Results

### 3.1 Gross Pathological Changes Induced by Fasciolosis in Cattle

Load of fasciolid flukes varied significantly, ranging from 2 to 328 numbers of *Fasciola* per infected bovine liver. The study was conducted between January 2021 to July 2025. The flukes were dorsoventrally flattened in appearance under microscope, distome (flukes having two suckers) with well‐developed cephalic cone and size varied from 5.0 × 1.0 to 2.1 × 0.8 cm (Supporting Fig. 1). Using the well‐established *pepck*‐based multiplex PCR, two types of fasciolid flukes had been confirmed in Bangladesh, such as pure (spermic) *F. gigantica* and parthenogenetic (aspermic) *Fasciola*. However, we could not find any *F. hepatica*. During *pepck*‐based multiplex PCR, spermic *F. gigantica* yield a single band at ~510 bp level whereas parthenogenetic *Fasciola* showed two bands ( ~ 510 bp and ~240 bp) (data not shown).

**Figure 1:**
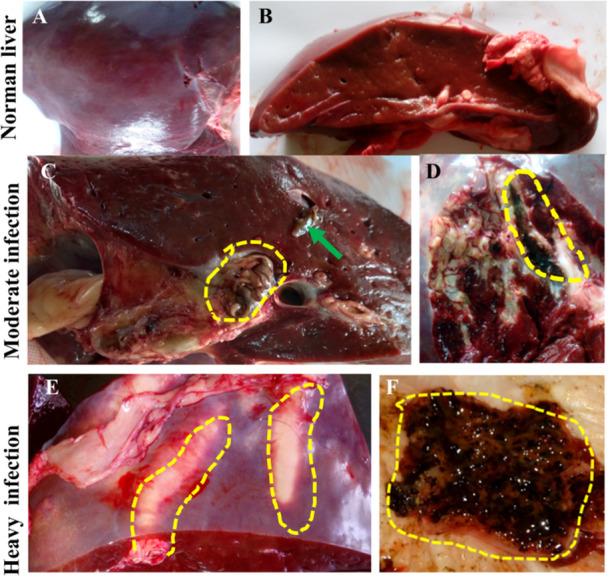
Normal and gross pathological changes in bovine liver induced by chronic fasciolosis. (A) Normal liver from the parietal surface. (B) Cross sections of normal liver. (C) Cross‐section of moderately affected liver. Yellow circle indicates crawling out of adult flukes. Arrow indicates aggressions of adult fluke. (D) Opened bile ducts showing whitish areas of fibrosis. Yellow circle indicates moderate deposition of blackish calcium salts. (E) Thickened and prominent bile ducts visible from the surface. Yellow dotted lines indicate the prominent bile ducts. (F) Pipe‐stem liver. Dotted yellow area shows the deposition of calcium salts.

We categorized the livers on the basis of induced pathologies into three types: normal (non‐infected), mild, and severely affected liver. Normal (non‐infected) bovine livers appeared as reddish‐brown color with a shiny capsule (Figure 1A,B). In mild cases, affected livers were characterized by the presence of parasites, moderate calcification and fibrosis only limited to the periphery of the bile ducts. The severely affected livers were characterized by thick, prominent bile ducts along with heavy calcification. Lumen of the bile ducts became so dark with heavy deposition of dark brown salts and was almost obliterated. During digital palpation, firm, solid masses were detected. When cut with a knife, they emitted metallic sounds and exhibited a granular appearance. Moreover, parasites emerged from the exposed surfaces. In chronic fasciolosis, the liver became atrophied with marked whitish patches of fibrosis (Figure 1C‐F). We also found heavy calcium salt deposition in the gallbladder (data not shown).

### 3.2 Massive Cirrhosis due to Fasciolosis in Cattle, Evident in Histopathology

**Figure 2:**
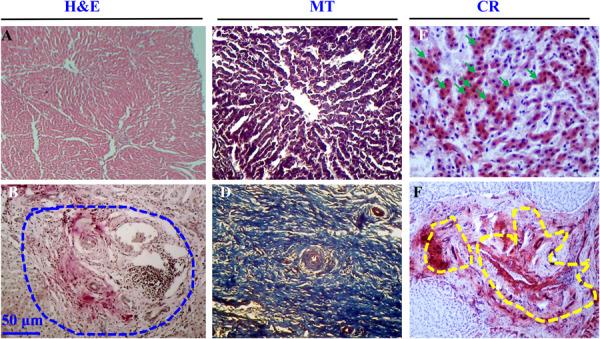
Histological analysis of the normal and *Fasciola gigantica* infected bovine livers. (A) Normal liver section stained by H&E. (B) Infected liver section stained by H&E. Blue circle indicate severely affected parts infiltrated with huge inflammatory cells. (C) Normal liver section stained by MT. (D) Infected liver section stained by MT. (E) Eosinophils stained by CR. Arrowheads indicate eosinophils. (F) Amyloid stained by CR, represented in yellow dotted areas. MT, Masson's trichrome; CR, Congo red.

For histopathology, thin sections from the infected and normal uninfected tissues were prepared and stained with H&E, CR and MT stains. During microscopic examinations of H&E stained sections, the normal architecture of the liver was almost replaced by the extensive fibrosis particularly in case of heavy infection. There was a massive proliferation of fibrous connective tissues around the bile ducts, resulting thickening of the wall of the bile ducts. Desquamation of bile duct epithelial cells was also evident (Figure 2A,B).

On the other hand, MT staining revealed that fibrosis started from bile ducts, later spread radially and gradually to the hepatic lobules. The proliferating fibrous tissues gradually replaced the normal hepatocytes, the building blocks and functional unites of liver. Such changes were not seen in the age and sex matched non‐infected (control) liver tissues (Figure 2C,D).

In addition, there were massive infiltrations of inflammatory cells in the affected tissues, which were predominantly mononuclear cells and polymorphonuclear cells resembling eosinophils. Histopathological analysis using CR revealed infiltration of cells containing intense red (pink) cytoplasmic granules and multi‐lobulated nucleus, confirming the infiltration of eosinophils (Figure 2E). Quantitative histopathological analysis showed that number of infiltrated eosinophils reached up to 57.9 ± 4.5 cells/focus (Data not shown). CR‐specific pink colored staining was also detected in the acellular areas, indicating deposition of amyloid in the liver tissues, particularly in the severely affected livers (Figure 2F).

### 3.3 Expression of GATA‐3 and Its Localization in the Zone of Inflammation

**Figure 3:**
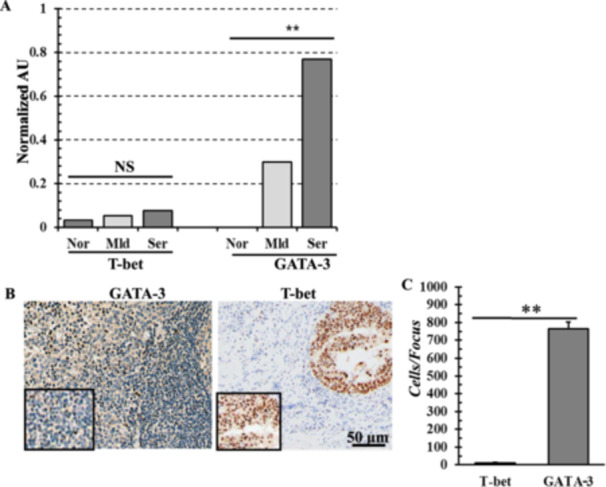
Quantification of transcription factors. (A) Expression of T‐bet and GATA‐3 at mRNA level. NS, non‐significant; Nor, normal; Mld, mild; Ser, severe. (B) Immunohistochemistry showing infiltration of T‐bet and GATA‐3 producing cells. T‐bet, T‐box expressed in T cells proteins; GATA‐3, GATA‐binding protein 3. (C) Quantification of T‐bet and GATA‐3 producing cells. **, *p* < 0.01.

Liver samples were collected from each group of mild (*n* = 10) and severely (*n* = 10) infected livers for the collection of total RNA. Total RNA extracted from non‐infected livers (*n* = 10) served as control. The sqRT‐PCR analysis showed that transcription factor such as GATA‐3 (296 fold) were highly expressed in severely infected livers. In contrast, T‐bet (2 fold) remained in basal level (Figure 3A). However, both transcription factors were either undetectable or expressed at basal levels in the livers of non‐infected control animals. In the same samples, GAPDH expression remained consistent across all cases, indicating uniform sample processing and reliable cDNA input for each reaction. To further validate the production of GATA‐3, immunohistochemistry was conducted using histological section of infected liver tissues. The study revealed the accumulation of a cluster of GATA‐3‐expressing cells in the infected liver. Importantly, very few T‐bet positive cells were detected. GATA‐3 positive cells were significantly (0 < 0.01) higher (765 ± 38) compared to T‐bet expressing cells (Figure 3B,C).

### 3.4 Confirmation of *F. gigantica‐*Induced Polarization of Immunity

**Figure 4:**
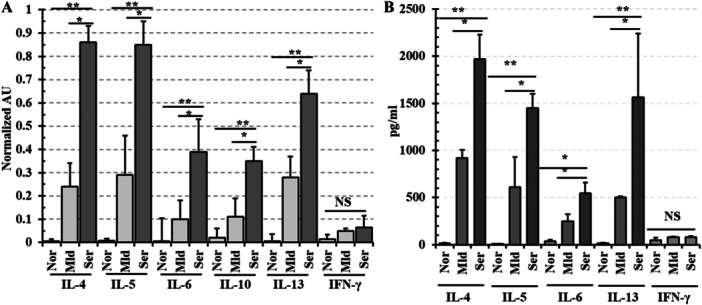
Quantification of cytokines. (A) Expression of cytokines at mRNA level. (B) Production of different cytokines as estimated in liver lysate by ELISA. NS, non‐significant; Nor, normal; Mld, mild; Ser, severe. *, *p* < 0.05; **, *p* < 0.01.

To confirm the Th2 shift of *Fasciola* induced immunity, IL‐4, IL‐5, IL‐6, IL‐10, IL‐13, and INF‐γ were quantified both at mRNA and protein levels. The sqRT‐PCR analysis indicated significantly elevated expression of mRNA of IL‐4, IL‐5, IL‐6, IL‐10, and IL‐13 in the severely affected livers, whereas expression of INF‐γ was not notably elevated. The levels of IL‐4, IL‐5, IL‐6, IL‐10, and IL‐13 were increased by 172‐fold, 106‐fold, 78‐fold, 16‐fold, and 106‐fold, respectively in infected groups as the expression levels were quantified relative to the corresponding transcript abundance in the non‐infected control group (Figure 4A). However, all cytokines were either undetectable or expressed at basal levels in the livers of non‐infected control animals. In the same samples, GAPDH expression remained consistent across all cases.

To further determine the bias of the host immune response—whether towards a Th1 or Th2 profile, total proteins (liver lysate) were extracted by homogenizing the liver samples of each group of control (*n* = 10), mild (*n* = 10) and severely (*n* = 10) affected livers. Levels of soluble cytokines like IL‐4, IL‐5, IL‐6, IL‐13, and INF‐γ were estimated by using Sandwich ELISA. IL‐4, IL‐5, IL‐6, and IL‐13 levels increased in both mild and severely affected livers. Soluble IL‐4 (1970 ± 258), IL‐5 (1448 ± 150), IL‐6 (544 ± 111) and IL‐13 (1563 ± 675) were significantly (*p* < 0.01) higher in severely infected samples as compared to the controls. Also, cytokine levels in severely affected livers were significantly higher (*p* < 0.05) compared to mildly affected cases. In contrast, INF‐γ production remained at basal levels in both mild and severely infected cattle livers (Figure 4B).

**Figure 5:**
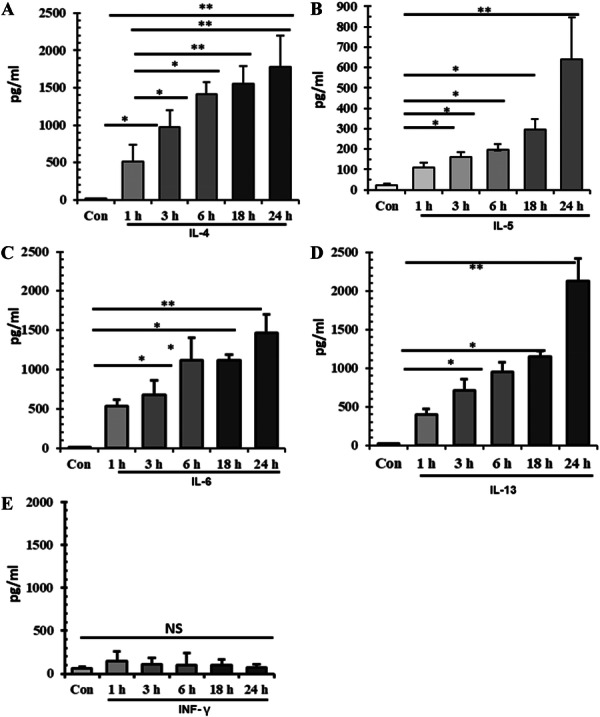
Quantification of cytokines in the culture media of bPBMCs stimulated with FCM. Production of different cytokines as estimated in liver lysate by ELISA. (A) Production of IL‐4 by bPBMCs in a time‐dependent manner. (B) Production of IL‐5 (C), Production of IL‐6, (D) Production of IL‐13, (E) Production of INF‐γ. NS, non‐significant; *, *p* < 0.05; **, *p* < 0.01.

To reinforce the validation of Th2‐biased immunity, bPBMCs were stimulated with FCM and measured various cytokines, which revealed elevation of IL4, IL‐5, IL6, and IL‐13 in a time dependent manner. Production of IL4 (1776.1 ± 423 pg/mL), IL‐5 (639.6 ± 208 pg/mL), IL6 (1471 ± 230 pg/mL) and IL‐13 (2126 ± 289 pg/mL) was significantly (*p *< 0.01) higher at 24 h of stimulation (Figure 5A‐D) by FCM treated cells compared to control bPBMCs. However, production of INF‐γ by FCM treatment of bPBMCs at basal level (76 ± 32 pg/mL) throughout the incubation period (Figure 5E).

## 4 Discussion

The liver is one of the body's most vital organs, essential for maintaining the overall health of the body. When liver flukes start residing there, they manipulate the host's immune response to support their own survival and growth. In doing so, they ‘re‐tune’ the immunological responses, dampening some defenses while redirecting others. This delicate immunological interference often allows the parasites to persist silently for longer periods [41]. Here, we present gross and histological changes, cytokines and transcription factor profiling along with detection of inflammatory cell populations.

Severe infection caused massive damages in the liver. Although detail pathological consequences are yet to be described in *F. gigantica* infection in cattle, however, pathogenesis and pathologies induced by *F. hepatica*, a very closely related fasciolid fluke, are well documented. Excretory and secretory (E/S) products of helminths have been proved to elucidate immune reactions within the host [42]. Considering the fact, we stimulated bPBMC with FCM and as expected we observed that FCM significantly elevated Th2‐mediated cytokines in cell culture milieu, providing unambiguous proof of Th2 drift immunity in the course of *F. gigantica* infection in cattle. Penetration of the immature flukes through liver parenchyma and localization of the adult flukes within the bile ducts exert a traumatic action resulting in a series of changes in the liver parenchyma [25]. During migration, the immature flukes caused severe tissue damage, resulting in hepatomegaly and hemorrhagic track formation. Later, the severely damaged hepatocytes were further replaced by proliferated fibrous tissues, changing the normal liver architecture. In addition, adult flukes residing within the lumen of the bile ducts exert pressure on the wall of the bile ducts, resulting in damage of the hepatocytes adjacent to the bile ducts. Damaged hepatocytes were replaced by the fibrous tissues that initiated biliary cirrhosis or sclerosing fibrosis [43]. Due to extensive fibrosis, the entire liver became atrophied and the biomass of the severely affected organ was drastically reduced; the edges of the liver became sharp and Glisson's membrane showed wrinkling. Waste products of parasites along with dead and denuded tissues deposited in the lumen of the bile ducts. Due to deposition of black materials in the lumen of the bile ducts, it looked like a clay pipe and the condition is popularly known as ‘pipe stem liver’ [44]. Our current study has also revealed the formation of pipe stem liver and heavy deposition of calcium salts, along with liver cirrhosis and atrophy, indicating that the pathological consequences of *F. gigantica* and *F. hepatica* are almost identical.

During histological examination, we observed that the normal histological architecture of the liver, consisting of hepatocytes, was almost replaced by extensive fibrosis, particularly in severe infection. Any affected organ tends to restore its damaged tissues in two ways: (i) minor losses of functional tissues by its own parenchymatic tissues, which is regeneration and (ii) massive losses of tissue by inducing proliferation of fibrous connective tissues, which is known as repair. In this study, we unambiguously proved the excessive proliferation of fibrous connective tissues by MT stain which is used as the gold standard for the confirmation of deposition of fibrous connective tissues irrespective of species [45]. There was massive infiltration of inflammatory cells in infected tissues, predominantly mononuclear cells and eosinophils. Eosinophils were confirmed by CR stain, which is commonly used to ensure the presence of eosinophils [46]. Among the inflammatory cells, eosinophils are associated with the immobilization and killing of indigestible large pathogens such as helminths and ectoparasites. Activated eosinophils entrap these large pathogens and kill them by various toxic granule proteins [47, 48, 49]. In addition, CR staining also revealed a huge deposition of amyloid in the acellular areas of some severely affected liver tissues. Amyloidosis is a rare condition that results from the abnormal extracellular deposition of amyloid (proteolytic‐resistant) protein in various vital organs such as brain, liver, heart, kidney, spleen and other parts of the body. Then they gradually replace the functional unit or parenchymatic cells of that organ, and impair the normal functions of the affected organs. Deposition of amyloid proteins has been reported in many diseases such as Alzheimer's disease, cancer progression and liver cirrhosis [50]. To our knowledge, we report amyloidosis for the first time in fasciolosis.

In this study, we found that IL‐4, IL‐5, IL‐6, IL‐10, and IL‐13 production has significantly elevated both in mRNA and protein levels. IL‐4, IL‐5, IL‐6, IL‐10, and IL‐13 are important cytokines for controlling extracellular parasites. IL‐4 is known to have the greatest effect in inducing Th2 differentiation and the GATA‐3 transcription factor is a key regulator of Th2 polarization [51]. GATA‐3 trans‐activates the IL‐4 cassette and promotes the transcription of IL‐4 and IL‐13. It also inhibits Th1 responses and is repressed during Th1 development [52]. The present study revealed that the expression of T‐bet was reduced, whereas the expression of GATA‐3 was increased and that GATA‐3 was predominantly expressed in fasciolosis, displaying a Th2 drift at the transcriptional level. Th2 drift is one of the common mechanisms for immune escape and is closely related to initiation, development, metabolism, reproduction and survival of *Fasciola* spp [37]. Thus, correction of the imbalance of Th1/Th2 could be an important way for *Fasciola* immunotherapy.

Among different liver flukes, the immunological orchestra of *F. hepatica* has been the most extensively studied. As noticed in *F. hepatica* infection, the interaction between flukes. and the host is complex, triggering a mixed Th1/Th2 cytokine response. However, the infection predominantly induces a Th2‐biased immune profile, characterized by elevated levels of IL‐4, IL‐5, IL‐10, IL‐23, and IL‐13, which appears to support parasite survival within the host. The role of IL‐6 in fasciolosis remains incompletely understood, as it has been implicated in both promoting host immune responses and contributing to parasite persistence [53]. The role of IL‐6 has also been associated with the onset of organ fibrosis [54]. The presence of IL‐6 in the *Fasciola‐*infected liver may have a profound impact on the pathogenesis of the extensive fibrosis in liver tissues [55]. However, an experimental infection with *F. gigantica* showed a modest increase of Th2‐type immune cytokines during the early phase of infection and immunosuppression during chronic infection in buffaloes [28].

It would be better, if we could give artificial infection to the natural hosts (e.g., cattle) with metacercariae and elucidate the immunological symphony, orchestrating the pathological changes induced by the bovine liver flukes. However, artificial infection with any trematodes requires production of metacercariae, that can only be generated by giving infections to the laboratory reared snail colonies with miracidium (the first larval stage of trematodes). At present, we do not have specific pathogen free snail colonies in our laboratory. Alternatively, we have collected bPBMCs and stimulated with FCM to show the productions of different cytokines, playing critical roles in the Th1/Th2 immune orchestration.

## 5 Conclusions

Taken together, our results suggest that liver flukes caused massive damage in the infected livers, characterized by severe fibrosis, mononuclear cell and eosinophilic infiltration, and deposition of amyloid. The study revealed Th2‐biased immunological drift both at transcriptional and translational levels, as evident by the significantly higher expression of GATA‐3, IL‐4, IL‐5, IL‐ 6, IL‐10 and IL‐13 in the infected liver compared to non‐infected control. In line with the *in vivo* data, results of *ex vivo* study also showed that bPBMCs stimulated with FCM also secreted significantly higher levels of Th2‐regulating cytokines, providing solid proof of the development of Th2‐biased immunity in *F. gigantica* infection in bovines. Overall, the findings of this study provide a clear link among immune profiles, pathological alterations, and disease severity in the complex host‐parasite interactions in *F. gigantica* infections, highlighting a strong association between elevated Th2 cytokine responses and the progression of liver fibrosis. To our knowledge, this is the first report on foundational insights into the immunopathological profile of *F. gigantica* infection and its association with liver pathology in naturally infected cattle.

## Author Contributions

Conceptualization by A., M.A.A., J.P., and M.A.H.N.A.K.; data generation by M.H.A., S.S.L., M.S.H., N.N.S., A.R.D., M.M.A., and A.; S.S.L., A., M.H.A., M.S.H., U.R.I., R.P., J.P., and M.S.H. wrote draft; A., M.H.A., N.N.S., A.R.D., J.P., M.S.H., and U.R.I. analyzed data; A., M.A.H.N.A.K., J.P., M.S.H., N.N.S., M.M.A. and M.A.A. revised manuscript.

## Ethics Statement

Our experimental protocols were reviewed and approved by the Animal Welfare and Experimentation Ethics Committee of Bangladesh Agricultural University, Mymensingh [Approval number: AWEEC/BAU/2021(37)]. All the experimental procedures were conducted following the guidelines given by the ethics committee.

## Conflicts of Interest

The authors declare no conflicts of interest.

## Supporting information


**Supplemental Figure 1:** Livers were dissected along the course of the bile ducts. Flukes were isolated, washed and photographs were taken.

## Data Availability

All data generated or analyzed during this study are included in the manuscript.
