# Supplementary figures and images for: Liver Cirrhosis Caused by Food‐Borne Zoonotic Fasciola gigantica in Cattle in Bangladesh: Pathology and Immunological Orchestra
Source: Immun Inflamm Dis. 2026 Jan 8;14(1):e70320. doi: 10.1002/iid3.70320 (PMC12780880; doi:10.1002/iid3.70320)

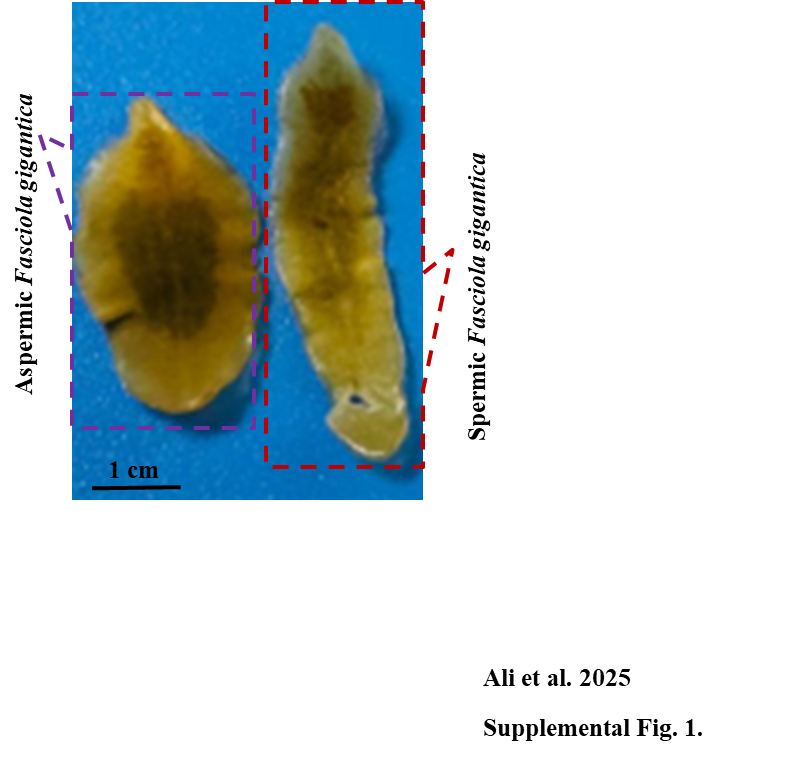

Supplement: Supplementary file 1 — Supplemental Figure 1: Livers were dissected along the course of the bile ducts. Flukes were isolated, washed and photographs were taken. [file IID3-14-e70320-s001.docx]
